# Band-and-wire pulley traction: endoscopic submucosal dissection of a gigantic gastric polyp aided by a novel adaptive traction technique

**DOI:** 10.1055/a-2387-3812

**Published:** 2024-09-20

**Authors:** Andrea Sorge, Maria Eva Argenziano, Pieter Jan Poortmans, Lynn Debels, Gian Eugenio Tontini, Maurizio Vecchi, David J. Tate

**Affiliations:** 1 Department of Pathophysiology and Transplantation, University of Milan, Milan, Italy; 2 Department of Gastroenterology and Hepatology, University Hospital of Ghent, Ghent, Belgium; 3 Clinic of Gastroenterology, Hepatology and Emergency Digestive Endoscopy, Università Politecnica delle Marche, Ancona, Italy; 4 Department of Gastroenterology and Hepatology, University Hospital Brussels (UZ Brussels), Brussels, Belgium; 5 Gastroenterology and Endoscopy Unit, Fondazione IRCCS Ca’ Granda Ospedale Maggiore Policlinico, Milan, Italy

An 85-year-old woman presenting iron deficiency anemia and refractory dyspepsia was referred due to a gastric lesion on the lesser curve of the corpus ( Video 1 ). The lesion was subpedunculated (Paris 0-Isp), 120 × 60 mm in diameter, with a short stalk and difficult access ( Fig. 1 ). To address the challenging accessibility during endoscopic submucosal dissection (ESD), a novel adaptive traction technique was employed ( Fig. 2 , Fig. 3 ).

Endoscopic submucosal dissection of a gigantic gastric polyp using the band-and-wire pulley traction technique.Video 1

**Fig. 1:**
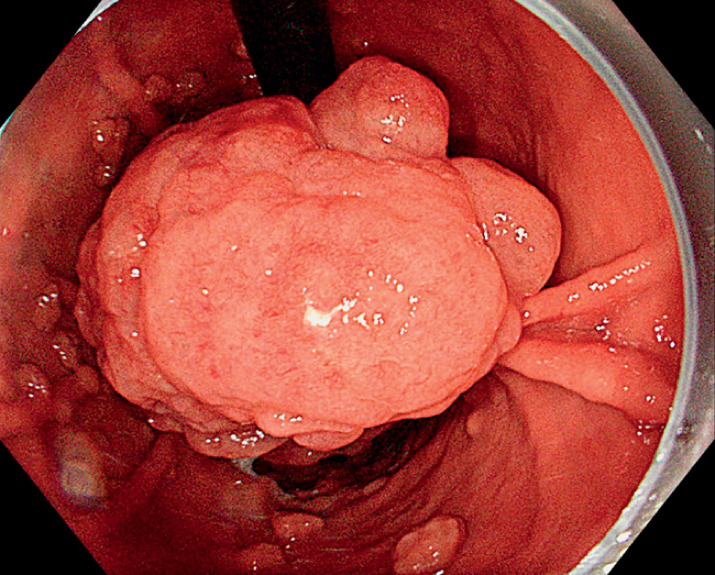
Bulky subpedunculated lesion on the lesser curve of the gastric corpus.

**Fig. 2:**
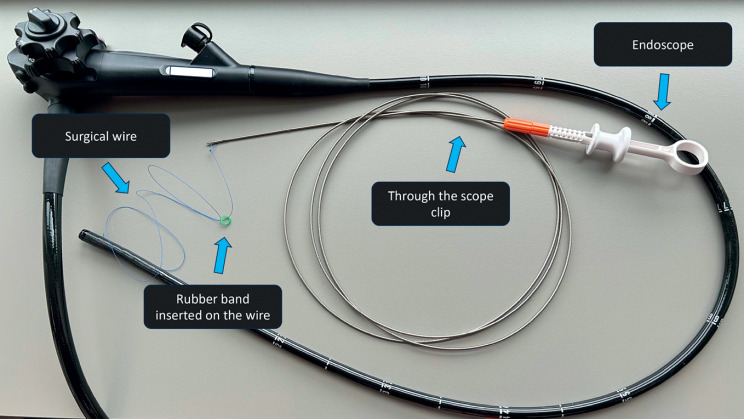
Adaptive band-and-wire pulley traction materials.

**Fig. 3:**
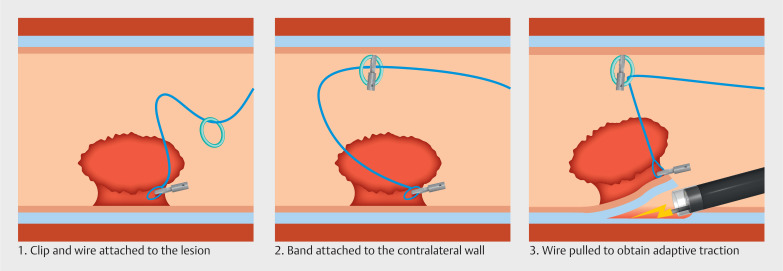
Schematic representation of the band-and-wire pulley traction technique.

A surgical wire with a loop at one end was clipped to the oral side of the polyp. Then, a band previously placed on the same wire was clipped to the contralateral gastric wall to provide traction for exposure of the stalk. After incision and tunneling, traction was applied on the anal side of the lesion through a second clipped wire. As dissection progressed, the traction was adapted by pulling the wires outside the patient to maintain optimal exposure of the submucosal plane. In total, three pulley traction devices were attached to the oral part of the lesion to complete the resection. The large size of the lesion precluded en bloc retrieval due to the risk of esophageal damage. Given the low suspicion of malignancy, the lesion was divided with a snare and retrieved. Closure of the resection site was not possible due to its size; three clips were placed centrally over the site of large vasculature divided during ESD.

There were no complications and the patient was discharged 24 hours after the resection. Histopathology revealed a nondysplastic hyperplastic polyp. At clinical follow-up 1 month after the resection, dyspepsia and anemia had resolved.

A novel band-and-wire pulley traction technique enabled safe and rapid ESD of a gigantic gastric polyp with difficult access. This technique allows the application of adaptive traction in any direction. The band-and-wire adaptive pulley traction technique enhanced access to the submucosal space and could be considered for gastric and rectal lesions with challenging access.

Endoscopy_UCTN_Code_TTT_1AO_2AG_3AD

## Notes

Endoscopy E-Videos https://eref.thieme.de/e-videos
*E-Videos* is an open access online section of the journal *Endoscopy* , reporting on interesting cases and new techniques in gastroenterological endoscopy. All papers include a high-quality video and are published with a Creative Commons CC-BY license. Endoscopy E-Videos qualify for HINARI discounts and waivers and eligibility is automatically checked during the submission process. We grant 100% waivers to articles whose corresponding authors are based in Group A countries and 50% waivers to those who are based in Group B countries as classified by Research4Life (see: https://www.research4life.org/access/eligibility/ ). This section has its own submission website at https://mc.manuscriptcentral.com/e-videos .

